# Analysis of Multiply Spliced Transcripts in Lymphoid Tissue Reservoirs of Rhesus Macaques Infected with RT-SHIV during HAART

**DOI:** 10.1371/journal.pone.0087914

**Published:** 2014-02-05

**Authors:** Jesse D. Deere, Robert C. Kauffman, Elda Cannavo, Joanne Higgins, Andradi Villalobos, Lourdes Adamson, Raymond F. Schinazi, Paul A. Luciw, Thomas W. North

**Affiliations:** 1 Center for Comparative Medicine, University of California Davis, Davis, California, United States of America; 2 Center for AIDS Research, Laboratory of Biochemical Pharmacology, Department of Pediatrics, Emory University School of Medicine, Veterans Affairs Medical Center, Atlanta, Georgia, United States of America; 3 Department of Pathology, School of Medicine, University of California Davis, Davis, California, United States of America; 4 Department of Veterinary Molecular Biosciences, University of California Davis, Davis, California, United States of America; University of California, San Francisco, United States of America

## Abstract

Highly active antiretroviral therapy (HAART) can reduce levels of human immunodeficiency virus type 1 (HIV-1) to undetectable levels in infected individuals, but the virus is not eradicated. The mechanisms of viral persistence during HAART are poorly defined, but some reservoirs have been identified, such as latently infected resting memory CD4^+^ T cells. During latency, in addition to blocks at the initiation and elongation steps of viral transcription, there is a block in the export of viral RNA (vRNA), leading to the accumulation of multiply-spliced transcripts in the nucleus. Two of the genes encoded by the multiply-spliced transcripts are Tat and Rev, which are essential early in the viral replication cycle and might indicate the state of infection in a given population of cells. Here, the levels of multiply-spliced transcripts were compared to the levels of *gag*-containing RNA in tissue samples from RT-SHIV-infected rhesus macaques treated with HAART. Splice site sequence variation was identified during development of a TaqMan PCR assay. Multiply-spliced transcripts were detected in gastrointestinal and lymphatic tissues, but not the thymus. Levels of multiply-spliced transcripts were lower than levels of *gag* RNA, and both correlated with plasma virus loads. The ratio of multiply-spliced to *gag* RNA was greatest in the gastrointestinal samples from macaques with plasma virus loads <50 vRNA copies per mL at necropsy. Levels of *gag* RNA and multiply-spliced mRNA in tissues from RT-SHIV-infected macaques correlate with plasma virus load.

## Introduction

Human immunodeficiency virus type 1 (HIV-1) persists in infected persons despite advancements in treatment. Current treatment regimens, called highly active antiretroviral therapy (HAART), usually consist of combinations of three or more drugs from two or more classes of antiretroviral agents (reviewed in [1]). HAART prevents progression to acquired immune deficiency syndrome (AIDS) in many individuals who are able to obtain and adhere to treatment regimens (reviewed in [1]). However, intermittent detectable viremia and viral rebound upon cessation of HAART demonstrate that the virus is not eradicated [2]–[6]. More sensitive virus load assays have demonstrated that low-level viremia persists during HAART [7]–[10].

The sources of persistent virus and residual viremia have been termed reservoirs, which have not been fully characterized. Several mechanisms could be contributing to viral persistence, including low-level viral replication or the persistence of long-lived cells that release virus slowly or episodically [7], [11], [12]. Two examples of long-lived cellular reservoirs that persist despite HAART are macrophages and resting memory CD4^+^ T lymphocytes [13]–[16]. Latently-infected resting memory CD4^+^ T cells contain a provirus predicted to be replication competent but express very little viral RNA (vRNA), resulting in non-productive infection [17], [18].

Although the exact mechanisms of viral latency have not yet been elucidated, several transcription-based models have been proposed [17], [19]. Silencing of the provirus may occur if the viral LTR is bound in inactive chromatin [20]–[24]. Another block to viral transcription is overcome by the viral protein trans-activator of transcription (Tat), which is encoded by multiply spliced viral mRNA (MS mRNA) [25], [26]. Tat is essential for viral replication [27], [28] and serves as a transcriptional anti-terminator [29] to increase the steady-state levels of viral mRNA [30]. The viral protein Rev is another essential viral protein encoded by MS mRNA [31], [32]. Rev binds to the Rev-response element (RRE) of full-length and partially spliced vRNA and plays a role in its transport from the nucleus to the cytoplasm for translation and packaging into virions [33], [34]. Interestingly, in purified resting memory CD4^+^ T cell populations from HAART-treated people an abundance of MS viral mRNAs have been detected despite an absence of cellular activation [35], [36]. This suggests that blocks at the transcriptional initiation and elongation steps are not absolute and that some transcription occurs, even in latently infected cells [35], [36].

Non-human primate animal models of AIDS have been developed and are being used to address viral persistence during HAART [37]–[41]. Due to the specificity of the non-nucleoside RT-inhibitors (NNRTIs) for HIV-1 reverse transcriptase (RT), some of these models use RT-SHIV [37]–[39]. RT-SHIV is a chimeric virus consisting of simian immunodeficiency virus mac239 with the reverse transcriptase of HIV-1 HXBc2 [42], [43]. Because RT-SHIV infection of macaques models HIV-1 infection in humans during HAART, it can be used to address questions of lentiviral persistence that are not feasible in human studies.

Recently, our group analyzed the distribution of RT-SHIV DNA and *gag* RNA in tissues collected at necropsy from macaques treated with HAART [37]. Analysis of whole tissue lysates and single-cell suspensions generated from those same tissues demonstrated that viral DNA (vDNA) and gag RNA were widely distributed in many tissues [37]. The results also suggested that some gag RNA was lost during the preparation of the single-cell suspensions [37]. It is not known whether this lost gag RNA was present in infected cells that were lost during preparation of the single-cell suspensions or contained in extracellular virions that were washed away during the preparation of the single-cell suspensions [37]. A previous study demonstrated that follicular dendritic cells (FDCs) in tissues in SIV-infected macaques can retain full-length vRNA, but not MS mRNA [44].

In the present study, we addressed the possibility that MS transcripts might serve as indicators of viral persistence in tissues from RT-SHIV-infected macaques during HAART. Levels of MS transcripts might indicate newly infected cells because they are essential early in the viral replication cycle, before the temporal switch to production of full-length vRNA. MS mRNAs also accumulate in the nucleus of latently-infected resting memory CD4^+^ T cells [35], [36]. We developed a two-step TaqMan real-time RT-PCR assay for the quantification of MS transcripts in tissues collected at necropsy. Levels of MS transcripts were compared with levels of *gag* RNA in the same tissues to determine whether productive viral infection or latency dominated in specific sites of viral persistence. Some of the macaques also received a regimen of valproic acid and prostratin as induction therapy designed to activate latent virus in the presence of HAART.

## Results

### Variation at the Exon-One and Exon-Two Splice Junction

**Figure 1:**
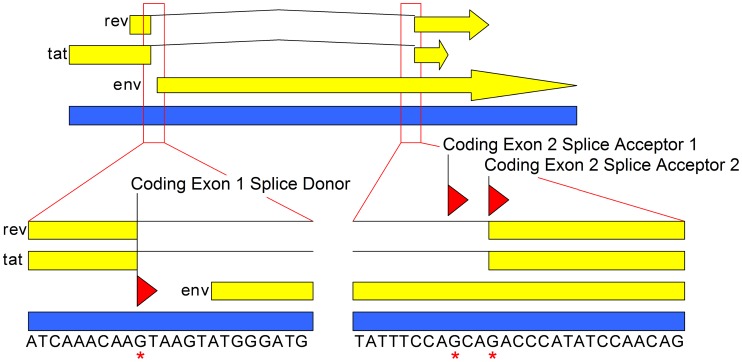
Map of the MS mRNA splice site. Genome map depicting the MS transcript region of RT-SHIV. There are two open reading frames on the MS transcript, encoding the Tat and Rev proteins. The Envelope protein (Env) is also encoded through this region. A portion of full-length viral sequence surrounding the splice junction is displayed (blue bar with sequence below). The splice donor and the alternative acceptor sites for the MS transcript are indicated with a red asterisk. A splicing event with splice acceptor 1 would result in a viral transcript containing the CAG sequence, while an event with splice acceptor 2 would result in a transcript without the CAG sequence.

The plasmid TOPOtat was generated by cloning the MS transcript from RT-SHIV infected CEMx174 cells into a TOPO vector to serve as a DNA standard in the development of a TaqMan real-time PCR assay for the quantification of the MS transcript. Upon sequence analysis, plasmid clones containing PCR-amplified MS transcripts from RT-SHIV-infected CEMx174 cells demonstrated sequence variation at the coding exon-one and coding exon-two splice junction (Table 1). The first variant contained an additional three nucleotides (+CAG) that were absent in the second variant (−CAG; Table 1 and Figure 1). A third, minor variant was observed which contained a C-to-T mutation (+TAG). While the viral envelope protein (Env) is also encoded in this region, the *env* mRNA is not spliced at this site. However, this codon is in frame and the observed C-to-T mutation would result in a stop codon (TAG) in the *env* reading frame.

**Table 1:** Variants found at the MS mRNA splice site^a^.

	% of Clones		
	−CAG	+CAG	+TAG
**CEMx174 cells**	31 (4/13)	46 (6/13)	23% (3/13)
**Jejunum**	14 (1/7)	86 (6/7)	0
**Spleen**	36 (4/11)	64 (7/11)	0
**Mes LN** b	60 (6/10)	40 (4/10)	0
**% of Total Tissue Clones** c	39 (11/28)	61 (17/28)	0

a Percent of plasmid clones displaying the indicated sequence at the coding exon-one and coding exon-two splice junction, number of clones in parenthesis.

b Mesenteric lymph node.

c Percentage of total cDNA clones obtained from jejunum, spleen, and mesenteric LN.

To determine whether this splice site sequence variation also occurred in animals, MS transcripts were PCR-amplified from several tissues from a monkey with a high plasma virus load (Mmu 36544; 1.29×10^6^ vRNA copies/mL) and subsequently cloned into a TOPO vector (Table 1). These plasmid clones also demonstrated sequence variation at the coding exon-one and coding exon-two splice junction, with some clones being variant one (+CAG) while others were variant two (−CAG; Table 1). None of the clones contained the +TAG variant.

Splice site sequence variation in the cloned MS transcripts could be the result of splicing event variation or mutations of either splice acceptor G-8803 or G-8805 in the viral genome (GenBank accession number M33262; Figure 1). The boundary region between the intron and the MS transcript coding exon-two was cloned into a TOPO vector and sequenced in order to determine whether the variation was the result of mutations in the viral genome (Table 2). Proviral DNA was amplified from peripheral blood mononuclear (PBM) cells and mesenteric lymph node tissue from animal Mmu 36544. Viral RNA was also cloned and sequenced from PBM cells and plasma. Five or six clones were sequenced for each sample to generate a total of 21 clones; all of these clones contained the wild type RT-SHIV sequence through the amplified region (Table 2).

**Table 2:** Sequence analysis of full-length viral genomes at the MS mRNA coding exon-two splice acceptor sites.

	Number of Clones	
	Wildtype^a^	Mutation^b^
**Plasma vRNA**	5	0
**PBM cell vRNA**	5	0
**PBM cell provirus**	5	0
**Mes LN** c **provirus**	6	0

a Wildtype sequence of RT-SHIV includes guanine at positions 8803 and 8805.

b A mutation at splice acceptor G-8803 would prevent the generation of a MS transcript containing the CAG sequence.

c Mesenteric lymph node.

Together, these results indicate that the observed sequence variation is the result of splicing event variation rather than mutations in the viral genome. These data also suggest that either of the guanine nucleotides at positions 8803 and 8805 can serve as coding exon-two splice acceptor sites, resulting in mRNA with or without the CAG sequence, respectively (Figure 1). Chi-squared analysis of the plasmid clones indicated a similar frequency in the number of transcripts generated from either splice acceptor site.

### Development of TaqMan PCR for Multiply Spliced Transcripts

**Figure 2:**
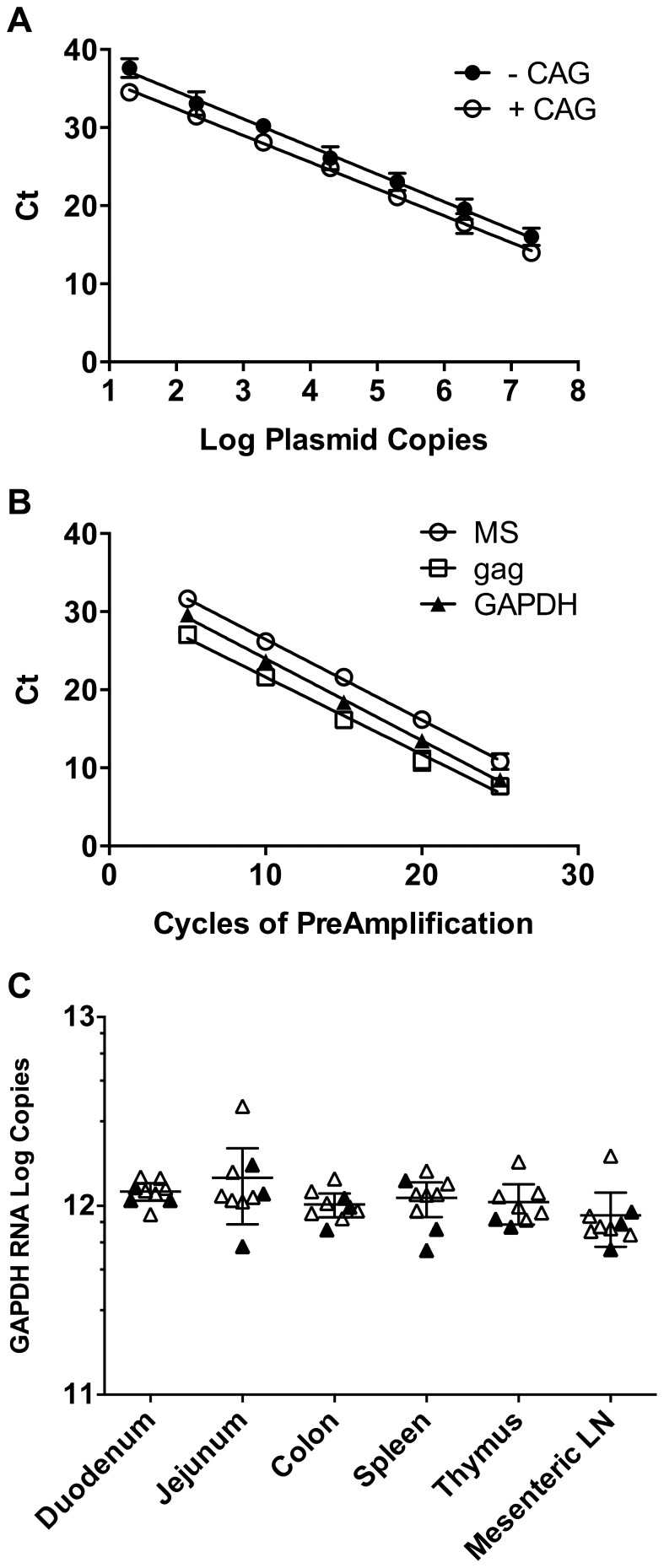
Validation of TaqMan PCR for MS mRNA, multiplex preamplification PCR, and distribution of *GAPDH* RNA in macaque tissues. A) Vectors containing a portion of the MS transcript with or without the CAG nucleotides at the coding exon-1 and coding exon-2 splice junction were used as standards for real-time TaqMan PCR. The plasmids were quantified by A260 spectrophotometric readings and serially diluted for quantification by TaqMan PCR. Data shown are the average threshold cycle (C_t_) +/− standard deviation of triplicate experiments, with some standard deviations too small to appear. The lines indicate regression analysis used to obtain line equations for calculating the amplification efficiencies and for the quantification of MS mRNA in samples. B) cDNA was generated from total RNA from RT-SHIV-infected CEMx174 cells isolated 48 hours post-infection using random hexamers primers. Multiplex preamplification PCR was performed for viral MS mRNA and *gag* RNA in addition to the cellular housekeeping gene *GAPDH* RNA. Individual reactions were removed from the cycler every five cycles, followed by quantification of the three transcripts by TaqMan PCR to determine whether the preamplification step was linear. Data shown are the average +/− standard deviation of triplicate experiments, with some standard deviations too small to appear. The lines indicate regression analysis. C) Total RNA was isolated from tissues and quantified by two-step TaqMan RT-PCR. Total cDNA was preamplified for 25 cycles in multiplex PCR before TaqMan PCR quantification. Cellular *GAPDH* RNA was measured in these six tissues from all nine macaques in order to determine whether it could serve as a standard. The solid symbols indicate samples from animals that received only HAART while the open symbols indicate samples from animals that received HAART plus induction therapy. The horizontal lines represent the averages with 95% confidence intervals.

TOPOtat was used for standardization of TaqMan real-time PCR in order to quantify the MS transcripts in tissues from RT-SHIV-infected macaques. Serial ten-fold dilutions of the plasmids TOPOtat-CAG and TOPOtat+CAG were used as templates in TaqMan PCR to generate standard curves. Results of TaqMan PCR using the SIVtat 284#2p probe (containing the CAG sequence) were linear over seven logs, whether the template contained the CAG nucleotides or not (Figure 2A). Linear regression analysis resulted in slopes of −3.54 for the –CAG template (r^2^ = 0.978) and –3.43 for the +CAG template (r^2^ = 0.989), indicating amplification efficiencies of 92% and 96%, respectively. Because this single set of TaqMan primers and probe detected both viral sequence variants efficiently, it was used for all subsequent analysis in this study.

Initial screening of six different tissues from RT-SHIV-infected, HAART-treated macaques demonstrated that MS mRNA levels were below the level of detection of standard TaqMan PCR in most samples (data not shown). To increase the sensitivity of the assay, a 25 cycle multiplex PCR preamplification step was developed. To test for linearity in the preamplification step, MS mRNA, *gag* RNA and cellular *GAPDH* RNA were amplified from total cDNA isolated from RT-SHIV-infected CEMx174 cells. Samples were removed from the cycler every five cycles, through 25 cycles. TaqMan PCR for each target was used in single-plex reactions to quantify copy numbers of RNA. Linear regression analysis demonstrated that the preamplification step was linear through 25 cycles for all three targets with r^2^ values for MS, *gag*, and *GAPDH* RNA of 0.993, 0.980, and 0.995, respectively (Figure 2B).

### *GAPDH* RNA Levels in Tissues

*GAPDH* RNA in six different tissues from each of the animals was quantified to determine whether this housekeeping gene could be used to normalize viral RNA content. Multiplex preamplification PCR was performed on cDNA generated from 4 µg of total RNA isolated from tissues from all nine macaques. TaqMan PCR of the preamplified cDNA demonstrated that *GAPDH* RNA levels were similar in all tissues (Figure 2C). A one-way analysis of variance (ANOVA) confirmed that the mean *GAPDH* RNA levels of the tissues were not significantly different. These results indicate that *GAPDH* RNA levels were consistent within this set of tissues, and therefore could serve for standardization purposes.

### Distribution of Viral RNA

Viral RNA was measured in plasma collected at necropsy from all nine animals (Tables 3 and 4). Three of these animals had plasma virus loads that were detectable by the standard virus load assay (>50 vRNA copies/mL; Table 3) [45]. The other six animals had plasma virus loads that were below the limit of detection of the standard virus load assay (<50vRNA copies/mL; Table 4). However, using a more sensitive virus load assay that utilizes ultracentrifugation to concentrate virus from plasma [46], low-level viremia was detected in three of these animals (Mmu 36348, Mmu 36166, and Mmu 36253; Table 4).

**Table 3:** MS mRNA and *gag* RNA analysis in macaques with plasma virus loads >50 vRNA copies/mL at necropsy^a^.

	Mmu 36544		Mmu 36160^b^		Mmu 36353^b^	
Plasma VL (vRNA copies/mL)	1,290,000		316		1,850	
	MS	gag	MS	gag	MS	gag
**Duodenum**	101	10,100	<0.06	7.1	2.0	60
**Jejunum**	55	13,700	<0.06	<0.06	<0.06	24
**Colon**	299	119,000	1.1	773	<0.06	11
**Spleen**	814	436,000	2.5	2,140	27	6,720
**Thymus**	<0.06	528	<0.06	2.0	<0.06	3.5
**Mes LN** c	357	103,000	<0.06	125	1.1	370

a Copies of either MS mRNA or *gag* RNA per 10^6^ copies of *GAPDH* RNA; <0.06 indicates that the sample was below the limit of detection.

b Also received induction therapy.

c Mesenteric lymph node.

**Table 4:** MS mRNA and *gag* RNA analysis in macaques with plasma virus loads <50 vRNA copies/mL at necropsy^a^.

	Mmu 36348		Mmu 36166		Mmu 36253^b^		Mmu 36661^b^		Mmu 36349^b^		Mmu 36488^b^	
Plasma VL (vRNA copies/mL)	11		25		5		<3		<50		<50	
	MS	gag	MS	gag	MS	gag	MS	gag	MS	gag	MS	gag
**Duodenum**	<0.06	5.8	3.9	19	<0.06	<0.06	2.5	40	<0.06	1.3	<0.06	1.6
**Jejunum**	<0.06	<0.06	<0.06	6.7	<0.06	<0.06	0.062	<0.06	<0.06	3.3	<0.06	8.6
**Colon**	<0.06	6.1	<0.06	18	3.83	39	<0.06	13	<0.06	<0.06	<0.06	584
**Spleen**	<0.06	1.8	<0.06	29	<0.06	18	<0.06	34	<0.06	24	0.11	104
**Thymus**	<0.06	<0.06	ND^d^	ND^d^	<0.06	6.2	<0.06	<0.06	<0.06	<0.06	<0.06	<0.06
**Mes LN** c	<0.06	41	<0.06	42	0.16	75	<0.06	49	<0.06	153	<0.06	282

a Copies of either MS mRNA or *gag* RNA per 10^6^ copies of *GAPDH* RNA; <0.06 indicates that the sample was below the limit of detection.

b Also received induction therapy.

c Mesenteric Lymph Node.

d Not Determined.

RT-SHIV MS mRNA and *gag* RNA were quantified by TaqMan real-time PCR as a measure of viral distribution in tissues during HAART (Tables 3 and 4). Due to the low levels of these transcripts on initial screening, the preamplification assay was used prior to TaqMan quantification. To standardize the values, copies of MS mRNA and *gag* RNA were normalized per 10^6^ copies of *GAPDH* RNA in each sample. Twenty-eight of the samples had detectable *gag* RNA, but no detectable MS mRNA (Tables 3 and 4). MS transcripts in tissues from macaques with plasma virus loads >50 vRNA copies/mL (Table 3) were detected more often than in animals with plasma viral loads <50 vRNA copies/mL (Table 4), 10 out of 18 samples *versus* 6 out of 35 samples, respectively. In two of the animals with plasma virus loads >50 vRNA copies/mL that received induction therapy, Mmu 36160 and 36353, the MS mRNA levels were below the level of detection in several samples. Levels of *gag* RNA in tissues displayed a similar pattern with generally higher levels detected in macaques with plasma virus loads >50 vRNA copies/mL than in those with plasma virus loads <50 vRNA copies/mL. MS mRNA was not detected in thymus samples from any of the macaques, despite detectable *gag* RNA in some (Mmu 36544, Mmu 36160, Mmu 36353, and Mmu 36253; Tables 3 and 4).

**Figure 3:**
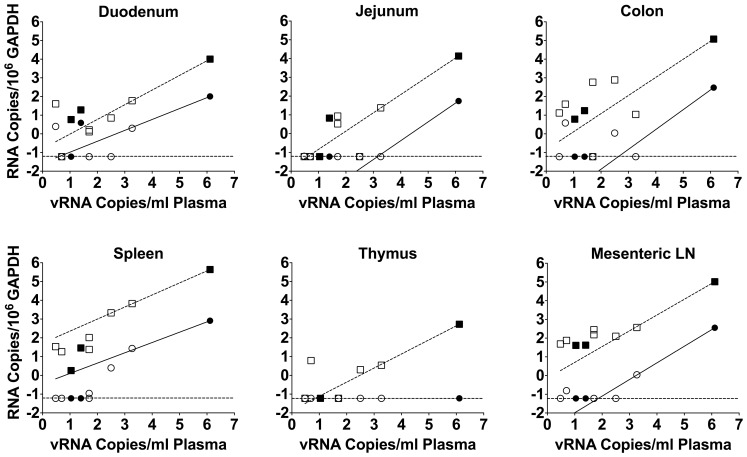
MS mRNA and *gag* RNA levels correlate with plasma virus loads in macaques. The correlations were determined between plasma virus loads and levels of the MS transcript (red circles) and *gag* RNA (blue squares) in tissues from infected macaques. The solid symbols indicate samples from animals that received only HAART while the open symbols indicate samples from animals that received HAART plus induction therapy. All nine animals from the study are represented in the figure. Both axes represent log RNA levels and the dashed line indicates the limit of detection.

To confirm the observation that higher plasma viral loads were associated with higher levels of RNA in tissues, correlations of MS mRNA and *gag* RNA to viral loads in plasma were determined (Figure 3). Correlations were significant (*P*<0.0001) for both MS mRNA and *gag* RNA for five of the tissues (duodenum, jejunum, colon, spleen, and mesenteric lymph node), with Pearson r values ranging from 0.9991 to 1.0000. Mmu 36544 had a plasma virus load at necropsy of 1.29×10^6^ vRNA copies/mL (Table 3). This plasma virus load is typical of untreated RT-SHIV-infected macaques [37], [38]. Nevertheless, we addressed the possibility that this single animal was over-influencing the correlations by excluding the data from that animal and re-assessing the correlations. While the re-assessed correlations observed for *gag* RNA to viral loads in plasma remained significant for all samples, the MS mRNA correlations were only significant for spleen and mesenteric lymph node samples (data not shown).

### Comparison of Gastrointestinal Tissue vRNA with Viremia

**Figure 4:**
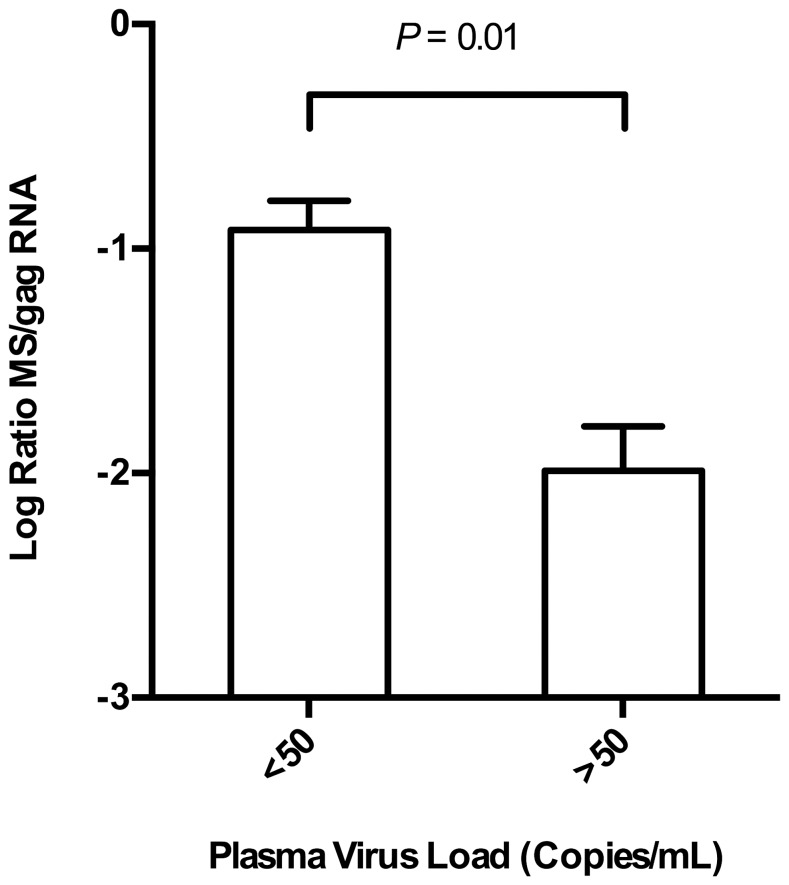
Ratio of MS mRNA to *gag* RNA in gastrointestinal tissues. The ratios of the MS transcript to *gag* RNA in duodenum, jejunum, and colon were calculated in order to determine whether there was an abundance of MS mRNA in tissues from macaques treated with HAART. Bars indicate average with standard error of the mean. Horizontal line indicates the results of an unpaired two-tailed student *t*-test.

To estimate whether tissues from animals treated with HAART displayed an abundance of MS mRNA relative to *gag* RNA, the ratios of MS mRNA levels to *gag* RNA levels were determined for samples that had detectable levels of both RNAs (Figure 4). This analysis was focused on duodenum, jejunum and colon samples because only 2 of 12 mesenteric lymph node and spleen samples had detectable levels of MS mRNA in macaques with well-suppressed plasma virus loads (<50vRNA copies/mL) at necropsy. An unpaired 2-tailed *t*-test demonstrated that the ratio of MS mRNA to *gag* RNA in the macaques with plasma virus loads <50 vRNA copies/mL was significantly higher than the ratio observed in animals with plasma virus loads >50 vRNA copies/mL (Figure 4, *P* = 0.01).

## Discussion

While it is apparent that HAART does not eradicate HIV-1 infection, reservoirs allowing for viral persistence during treatment are poorly understood. To help elucidate the mechanisms of viral persistence, we compared the distribution of viral MS mRNA and *gag* RNA in tissues from RT-SHIV-infected macaques treated with HAART. Due to the possibility that both low-level viral replication and long-lived cellular reservoirs might persist despite HAART, we elected to analyze total tissue lysates as opposed to populations enriched for resting memory CD4^+^ T lymphocytes [8], [12], [13], [47]–[49]. Enrichment of specific populations of cells might exclude other cells that are persistently infected or harboring virus. A previous study utilizing *in situ* hybridization in tissues from SIV-infected macaques demonstrated that *gag* RNA was more prevalent than MS mRNA in the absence of HAART [44]. The results presented here demonstrated that *gag* RNA was also present at higher levels than MS mRNA in tissues from HAART-treated, RT-SHIV-infected macaques.

During development of a TaqMan real-time PCR assay for quantifying MS transcripts in tissues from RT-SHIV-infected macaques, sequence variation at the MS mRNA coding exon-one and coding exon-two splice junction was identified. Analysis of genomic viral nucleic acids indicated that the observed splicing difference is a consequence of differential splicing and not due to mutations in the viral genome. Other groups have reported the presence of alternative splice acceptors or sequence variation at the coding exon-one and coding exon-two splice junction of MS mRNA in several strains of SIV and cell culture systems [50]–[55]. To our knowledge, our report is the first to describe this alternative splicing event *in vivo*. Our results from tissues support the general pattern of splice acceptor usage previously described in the cell culture experiments, with 39% of the 28 clones that we sequenced utilizing the first splice acceptor and 61% utilizing the second acceptor (Table 1). The functional effects of these variants on Tat or Rev are not clear. Tat proteins expressed from cDNA clones of the three variants observed by Viglianti *et al.* did not differ significantly in trans-activation of SIVmac LTRs [51]. Whether they differ in function *in vivo* is not known.

A similar pattern of three potential splice acceptors for coding exon-two of tat and rev mRNA is present in the genomes of HIV-1_HXB2_ [56] and HIV-1_NL4-3_ [57]. These splice acceptors were designated SA7, SA7a, and SA7b [56]. Purcell *et al.* demonstrated that only one of these three splice acceptors (SA7) is used for coding exon-two of MS mRNA in HIV-1_NL4-3_-infected cells [57]. It is not known whether other strains of HIV-1 utilize any of the other splice acceptors.

The third variant that we observed in cDNA clones generated from RT-SHIV-infected CEMx174 cells was not observed in any of the clones generated from tissues. Kodama *et al*. previously reported on the presence of premature stop codons in the transmembrane domain of SIV *env* [58]. Our results support their conclusion that the TAG variant is an *in vitro* artifact due to SIV replication in unnatural human cells.

TaqMan quantification of MS mRNA from the HAART-treated macaques indicated that no tissue had consistently detectable levels of MS mRNA. Interestingly, MS mRNA was not detected in any of the thymus samples despite detectable levels of *gag* RNA in some. Because the *gag* RNA levels that were detected were at lower levels than in the other tissues, it is possible that viral replication or reactivation from latency is occurring in the thymus but at levels too low to detect the MS mRNA. However, the lack of MS mRNA suggested that the detected *gag* RNA in thymus might be virion-associated or trapped in cells such as FDCs [44]. These results support a previous study that concluded that thymocytes are not a viral reservoir in SIV-infected pig-tailed macaques treated with antiretroviral therapy [59].

MS mRNA and *gag* RNA levels from well-suppressed animals (<50 vRNA copies/mL) were compared with the levels observed in animals with higher virus loads (>50 vRNA copies/mL). While none of the tissues contained an abundance of MS transcripts relative to *gag* RNA, the gastrointestinal samples from well-suppressed macaques had a significantly higher ratio of MS to *gag* RNA, which suggests a state of viral latency in these samples. Two recent studies by Yukl *et al.* analyzed biopsies taken from gastrointestinal compartments of HIV-1-infected patients during HAART [60], [61]. In those studies, MS mRNA was not detected in the gastrointestinal samples, but a preamplification step was not employed [60], [61]. However, biopsies taken from the large bowel tended to have lower ratios of *gag* RNA to vDNA, suggesting viral latency in these tissues despite high numbers of activated CD4^+^ T cells [60], [61]. Their results also suggested that the ileum tended to have higher ratios, indicating active replication [60], [61]. We did not analyze ileum samples, but our results support their conclusions that the other regions of the gut might be a potential reservoir of latent virus during HAART. The preamplification step that was utilized in our study enabled the detection of low-level MS mRNA. A future study designed to analyze specific cell-types for MS and *gag* RNA content using a preamplification step may help to further elucidate the state of infection in these tissues.

An accumulation of MS mRNA in several key lymphoid tissues was not observed despite the essential role of MS transcripts early in the infection cycle and evidence that MS mRNAs accumulate in the nucleus of latently-infected cells [35], [36]. The levels of MS mRNA in tissues from these macaques were very low, many below the level of detection. *gag* RNA, which serves as genomic vRNA as well as mRNA for Gag and Pol proteins, was present at higher levels in these tissues. Importantly, while *gag* RNA is present in virions and can persist in FDCs in the absence of infection [44], MS mRNA is a product of the splicing machinery within infected cells. In RT-SHIV-infected rhesus macaques, levels of both MS mRNA and *gag* RNA in tissues correlate with plasma virus load.

## Materials and Methods

### Animals and Infection

Nine juvenile, male Rhesus macaques (*Macaca mulatta*; Mmu) from the retrovirus-free colony of the California National Primate Research Center (CNPRC) were used. This study was approved by the Association for the Assessment and Accreditation of Laboratory Animal Care, International (AAALAC) accredited University of California, Davis Institutional Care and Use Committee (IACUC). The UC Davis IACUC has an Animal Welfare Assurance on file with the Office of Laboratory Animal Welfare (OLAW). Animals were administered 10 mg/kg body weight ketamine-HCl (Parke-Davis, Morris Plains, NJ, USA) intramuscularly when necessary for immobilization. Additionally, analgesics were administered at the discretion of the CNPRC veterinary staff in an effort to minimize all animal pain and discomfort. Macaques were housed at the California National Primate Research Center, which is fully accredited by the Association for the Assessment and Accreditation of Laboratory Animal Care (AAALAC). For housing, animals were maintained in cages with 4 square feet of floor space, or 6 square feet if over 10 kg, and fixed perch bars in a temperature-controlled BSL-2+ vivarium with continuous monitoring of temperature and humidity. Compatible animals were paired continuously or intermittently (separated at night) whenever possible. All animals had visual and auditory access to other macaques 24 hours per day. These animals were fed a balanced commercial macaque chow (Purina Mills, Gray Summit, MO) twice daily and fresh produce twice weekly, with free access to water 24 hours per day. Supplemental food was provided when clinically indicated. Environmental enrichment was provided daily, included manipulanda (forage boards, mirrors, puzzle feeders) and novel foodstuffs.

The endpoint of this study was set at a pre-specified timepoint as part of the experimental design of the antiretroviral treatment regimen (see Antiretroviral Drugs and Induction Treatment). All macaques were humanely euthanized by overdose of sodium pentobarbital (60 mg/kg) administered by the intravenous route under ketamine sedation (10 mg/kg).

For infection of animals, stocks of RT-SHIV were prepared from cell culture supernatants of CEMx174 cells transfected with the two viral half-clone plasmids as previously described [38], [62]. Prior to infection, virus stocks were sequenced and contained the required T-to-C substitution at position 8 of the SIV tRNA primer binding site [63]. Macaques were inoculated intravenously with 10^5^ 50% tissue culture infectious doses of RT-SHIV as previously described [46].

### Antiretroviral Drugs and Induction Treatment

RT-SHIV-infected rhesus macaques were treated with HAART as previously described [37]. Briefly, all nine macaques were treated with a HAART regimen beginning six weeks post-infection, consisting of tenofovir (PMPA), emtricitabine (FTC), and efavirenz (EFV; Sustiva). FTC and PMPA were provided by Gilead Sciences (Foster City, CA, USA) and EFV was purchased from a pharmacy. PMPA and FTC were administered subcutaneously, once daily. FTC was reconstituted in phosphate buffered saline (pH 7.4) and administered at 16 mg/kg body weight. PMPA was re-suspended in distilled water, buffered with NaOH to pH 7.0 and delivered at 30 mg/kg body weight. EFV was delivered orally at 200 mg per day in food, as previously described [37], [38]. Animals were monitored weekly for weight and the drug doses were adjusted according to body weight. After 15 weeks of therapy, the dose of PMPA was reduced to 15 mg/kg body weight to avoid renal toxicity [64].

After 26 weeks of HAART, six of the HAART-treated macaques were treated with prostratin and valproic acid for eight weeks in addition to continuing HAART. During the fist two weeks of the induction period, each animal received induction treatment five times. Over the final six weeks of the induction period, each animal received weekly treatment. Prostratin (Salvia Sciences, Palo Alto, CA) was delivered to anesthetized animals at 0.1 mg/kg by infusion in saphenous or cephalic veins. Valproic acid (Upsher-Smith Laboratories, Minneapolis MN) was delivered orally at 120 mg/kg in two doses, the first dose being delivered by orogastric intubation during anesthesia and the second being delivered in food later the same day. After the induction period, the animals were continued on HAART alone until euthanasia. Complete details of the induction treatment, including pharmacokinetics, will be published elsewhere (P.A. Luciw, manuscript in preparation).

After 41 weeks of HAART, four of the animals (Mmu 36348, Mmu 36166, Mmu 36253, and Mmu 36661) were sedated with ketamine-HCl and then humanely euthanized with a barbiturate overdose by veterinary pathologists and staff at the CNPRC. HAART was stopped in the remaining five animals (Mmu 36544, Mmu 36160, Mmu 36353, Mmu 36349, and Mmu 36488) and plasma vRNA was monitored for viral rebound. After 16–20 weeks without HAART, these animals were subsequently euthanized.

### Isolation of RNA

In order to protect RNA in tissues from degradation at necropsy, sections of tissue were treated with Qiagen RNA*later* RNA Stabilization Reagent (Valencia, CA, USA) according to the manufacturer’s protocol. Briefly, sections of tissue no greater than 0.5 cm per dimension were cut from tissues using sterile scalpels. Tissues were then stored in RNA*later* overnight at 4°C. The following day they were transferred to 1.5 ml centrifuge tubes and stored at −80°C until nucleic acid extraction.

Tissues were disrupted for isolation of RNA with QIAzol Lysis Reagent (Qiagen) using a Qiagen TissueRuptor (a rotor-stator homogenizer). Frozen samples were weighed and 700 µL of QIAzol per 50 mg of tissue was added. The samples were chopped with sterile scalpels and then disrupted using the TissueRuptor with autoclaved, disposable probes to prevent carry-over contamination. The lysates were separated into 700 µL aliquots before the addition of 150 µL of chloroform (Sigma-Aldrich, St. Louis, MO, USA), followed by 15 sec of vigorous shaking. After three minutes at room temperature, the samples were centrifuged at 12,000×g at 4°C. The aqueous phase was removed from each sample for RNA isolation and the remainder of each lysate was saved for DNA isolation (see below).

For RNA extraction, 1.5 volumes of molecular grade absolute ethanol (Sigma) was added to the aqueous phase and the samples were applied to Qiagen miRNeasy mini columns according to manufacturer’s protocol. Two sequential DNase digestions using the RNase-Free DNase Set (Qiagen) were performed on each sample according to the on-column treatment procedure. RNA was eluted from each column using 52 µL of molecular grade water. The optical density of each sample was measured using a spectrophotometer at 260 nm and the samples were diluted to 10 µg of RNA per 50 µL of molecular grade water. An additional DNase treatment was performed using TURBO DNase (Invitrogen, Carlsbad, CA, USA) according to the manufacturer’s protocol. The RNA was then stored in aliquots at −80°C.

Viral RNA was also isolated from 140 µL of plasma using the QIAamp Viral RNA Mini Kit (Qiagen) according to the manufacturer’s instructions. The RNA was eluted in molecular grade water and aliquots were stored at −80°C.

### Isolation of DNA

DNA was isolated from the fraction of QIAzol lysates not used for total RNA isolation. Purification of DNA was accomplished by precipitation of genomic DNA from the organic phenol/chloroform phase of each lysate by adding 0.3 volumes of molecular grade absolute ethanol (Sigma). Samples were centrifuged at 2000×g for five minutes at 4°C to pellet the DNA, and the supernatant was discarded. The DNA was washed twice with 700 µL of 0.1 M sodium citrate containing 10% ethanol with a centrifugation step after each wash as described above. Next, the DNA was washed with 75% ethanol, followed by another centrifugation step. The DNA was then dissolved in 500–1,400 µL of molecular grade water. The DNA solution was sheared using a Qiagen QIAshredder according to manufacturer’s instructions in order to reduce viscosity.

### Construction of TOPOtat

A portion of the RT-SHIV MS transcript was cloned into a vector to be used as a standard for the development of a real-time TaqMan PCR assay. As a source for the MS transcripts, CEMx174 were infected with RT-SHIV at a multiplicity of infection of 0.01. Total RNA was isolated from the infected cells 48 hrs post-infection using Qiagen RNeasy columns according to the manufacturer’s protocol. MS mRNA was amplified from the total RNA preparations in a one-step RT-PCR using Invitrogen SuperScript III Reverse Transcriptase (RT) and Platinum Taq DNA polymerase (Invitrogen) with 45 cycles of amplification and primers SIV tatF6616 and SIV tatR9157 (Table 5) at 200 nM each. Because the primers also have binding sites in the full-length viral genome, a short extension time of one minute was used to selectively amplify a portion of the MS transcript (332 base pairs) relative to the amplicon which would be produced from the viral genome (2541 base pairs). The PCR products were analyzed by agarose gel electrophoresis, and then cloned into pCRII-TOPO using TA cloning (Invitrogen) according to the manufacturer’s protocol to generate the vector TOPOtat. TOPOtat was propagated in TOP10F’ *Escherichia coli*, purified using a Qiagen Plasmid Maxi prep kit, and then the sequence was verified.

**Table 5:** Sequences of the PCR primers and the SIVtat 284#2p TaqMan probe.

Primer	Sequence (5′ → 3′)
SIVmac239 8006	TAACTCCACAGTGACCAGTCTCATAGC
SIVmac239 8510	CTAACACCAAAGTGGAACAATGAGAC
SIV tatF6616	CATGCATTTCAGAGGCGG
SIV tatR9157	TATCTGCCAAGGCCAGGAG
SIV MS PreAmpF6801	AGAAGAAGAACTCCGAAAAAGGC
SIV MS PreAmpR9127	GCCTTCTCCACCGTCTCTTTC
SIV MS TranF6814	CGAAAAAGGCTAAGGCTAATACATCT
SIV MS TranR9117	CCGTCTCTTTCTTTGCCTTCTC
SIVtat 284 #2p	aCTGCATCAAACAACAGACCCATATCCAACAG^b^

a FAM labeled nucleotide.

b TAMRA coupled nucleotide.

### Sequencing of Viral Nucleic Acids

Due to sequence variation in TOPOtat at the splice site of the MS transcript, viral cDNAs from various sources were cloned and sequenced to determine whether the variation was splicing variation or viral mutation. Preamplified cDNA from macaque tissues (prepared as described below) were amplified by nested PCR in order to generate amplicons suitable for TOPO-TA cloning and subsequent sequencing. The preamplified cDNA PCR product was diluted ten-fold and 1 µL was used as a template for second round PCR with Advantage 2 DNA Polymerase (Clonetech, Mountain View, CA, USA) using primers SIV MS TranF6814 and SIV MS TranR9117 (Table 5) at 250 nM each. Conditions for the second round reaction were: 95°C for 2 min, followed by 30 cycles of 95°C for 20 s, 55°C for 20 s, and 72°C for 20 s and ending with an extension step at 72°C for 10 min. Second round PCR amplification products were cloned into pCR2.1-TOPO (Invitrogen), propagated in *E. coli,* and sequenced.

Nested PCR was used to amplify a portion of unspliced full-length virus from plasma vRNA, RNA from infected PBM cells, and DNA from PBM cells and mesenteric lymph node samples from infected macaques. The region amplified for sequence analysis included a portion of the Tat/Rev encoding region and the intron, corresponding to bases 7980 through 8871 of SIVmac239 genome (GenBank accession number M33262) [65]. First round PCR was performed with primers SIVmac239 8006 and SIV MS PreAmpR9127 at a concentration of 250 nM, 0.05 U/µL REDTaq DNA Polymerase (Sigma), and approximately 1×10^6^ cell equivalents of genomic DNA or 20 µL of cDNA. First round reaction conditions were: 95°C for 2 min, followed by 25 cycles of 95°C for 20 s, 55°C for 20 s, 72°C for 1 min and a final extension step at 72°C for 10 min. Second round PCR was performed with primers SIVmac239 8510 and SIV MS TranR9117 and 1 µL of a ten-fold dilution of the first round PCR product. Second round reaction conditions were identical to those of the first round, except that a 30 second extension time was used and products were amplified for 30 cycles. PCR products were cloned into pCR2.1-TOPO (Invitrogen), propagated in *E. coli,* and submitted for sequence analysis at the UC Davis sequencing facility.

### Standardization of TaqMan PCR for MS Transcripts

TOPOtat was used as a standard for the development of a TaqMan PCR assay for the detection of the MS transcripts. The primers were designed to target both *tat* and *rev* mRNA. To avoid detection of full-length unspliced vRNA or viral DNA, the probe was designed to span the coding exon-one and coding exon-two splice junction of the MS transcripts. For standardization, plasmid DNA was quantified spectrophotometrically at 260 nm and copy numbers were determined. Ten-fold serial dilutions of this stock were used as templates in a 12 µL TaqMan PCR assay using Applied Biosystems (ABI) Universal Master Mix (Carlsbad, CA, USA) on an ABI 7900 cycler. The primers used were SIV MS TranF6814 and SIV MS TranR9117 at 400 nM and the TaqMan probe SIVtat284#2p was used at 80 nM (Table 5). Amplification efficiency (*E*) was determined as previously described [66] using the equation: 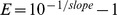.

### Multiplex PCR Preamplification and TaqMan Analysis

TaqMan PCR was used to quantify MS mRNA, *gag* RNA, and cellular *GAPDH* mRNA in total RNA from RT-SHIV-infected CEMx174 cells and in tissues collected from macaques at necropsy. cDNA was generated from total RNA using SuperScript III RT, random hexamers, and RNase inhibitor (Invitrogen) as previously described [37]. Due to the very low levels of viral transcripts in macaques treated with HAART, a multiplex preamplification PCR step was employed on the cDNA prior to TaqMan quantification [37], [67]. In the multiplex PCR, primers specific for MS mRNA were used, SIV MS PreAmpF6801 and SIV MS PreAmpR9127 (Table 5), in combination with primers specific for *gag* and *GAPDH* as previously described [37]. After 25 cycles of PCR using Advantage 2 DNA polymerase, the preamplification products were quantified using TaqMan PCR in single-target reactions (single-plex). The threshold cycles (C_t_) obtained from the TaqMan PCR analysis were used to determine the levels of MS mRNA using the standard equation obtained from the TOPOtat standard curve. Viral *gag* and cellular *GAPDH* RNA levels were also determined based on standard curves as previously described [37], [38], [45]. Because the preamplification was performed in multiplex reactions, the MS and *gag* RNA levels were normalized by the preamplified *GAPDH* RNA levels for standardization purposes.
